# Lacrimal Canalicular Wall Dehiscence/Thinning Found in Adults: A Case Series

**DOI:** 10.7759/cureus.58726

**Published:** 2024-04-22

**Authors:** Jonnah Kristina Teope, Steffani Krista Someda, Yasuhiro Takahashi

**Affiliations:** 1 Oculoplastic, Orbital, and Lacrimal Surgery, Aichi Medical University Hospital, Nagakute, JPN

**Keywords:** adults, thinning, epiphora, dehiscence, canalicular wall

## Abstract

We retrospectively reviewed the medical records of all patients diagnosed with lacrimal canalicular wall dehiscence/thinning from January 2020 to January 2024 and found three patients. Two patients were male, and the other patient was female. Patient ages ranged from 53 to 82 years. None of the patients had a history of ocular trauma, congenital anomaly, or other ocular diseases except for cataract. All patients complained of epiphora, and the duration of symptom ranged from 15 months to 10 years. Unilateral involvement of the lower canaliculus and isolated single wall involvement affecting only the canalicular roof were observed in all patients. The roof was dehiscent in one case and thinned out in the other two cases. The dehiscent canaliculus was closed by sutures, and the thinned-out canalicular wall of one patient was covered using a conjunctival flap. However, recurrences were noted at two and five months after surgery.

## Introduction

Canalicular wall anomaly is a rare cause of proximal lacrimal dysfunction and is conventionally considered congenital in etiology [1,2]. The most extensive study on canalicular wall anomaly, including seven dysgenetic canaliculi in pediatric patients, was conducted by Ali and Naik [2]. They postulated possible embryonic interferences that may cause aplasia or hypoplasia of the canalicular wall [2]. They further categorized aplasia and hypoplasia into single or multiple and focal or diffuse [2]. The canalicular wall was divided into four walls, namely, roof, floor, anterior wall, and posterior wall toward the conjunctiva [2].

Canalicular architecture may also be altered by trauma, infection, tumors, chemotherapeutic drugs, topical medications, and inflammation [1]. To the best of our knowledge, there is no published literature on lacrimal canalicular wall dehiscence and thinning found in adults. We herein present three adult cases of such canalicular wall dehiscence/thinning.

## Case presentation

This study was conducted in accordance with the tenets of the Declaration of Helsinki and its later amendments. This study protocol was reviewed, and the need for approval was waived by the institutional review board of our university hospital.

**Figure 1:**
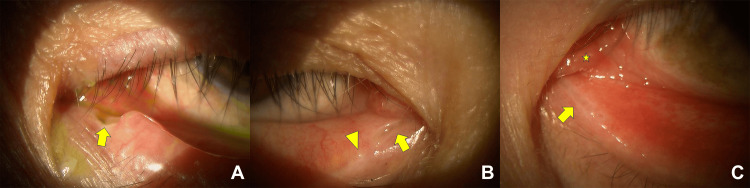
Slit-lamp photos A: case 1, B: case 2, C: case 3 Arrows indicate lacrimal canalicular wall dehiscence or thinning. The arrowhead indicates punctal obstruction, and the asterisk indicates caruncle and plica hypertrophy.

We retrospectively reviewed the medical records of all patients diagnosed with lacrimal canalicular wall anomaly from January 2020 to January 2024 and found three adult patients. Demographic and clinical data on these patients are shown in Table 1. Two patients were male, and the other patient was female. Patient ages ranged from 53 to 82 years. None of the patients had a history of ocular trauma, congenital anomaly, or other ocular disease, except for cataract in one case. All patients complained of epiphora, and the duration of symptom ranged from 15 months to 10 years. Unilateral lower canaliculus was involved in all patients, with the affected side being the right side in one patient and the left side in the other two patients. When the canalicular wall was divided into four walls as mentioned above [2], only the roof was involved in all patients. The roof was dehiscent in one case (Figure 1A) and thinned out in the other two cases (Figure 1B, 1C). One of the two patients with canalicular wall thinning was noted to have caruncle and plica hypertrophy (Figure 1C), while the other had concomitant upper and lower punctal obstruction on the affected side (Figure 1B). Tear meniscus height (TMH) measured using anterior segment optical coherence tomography (AS-OCT) was 209-616 µm on the affected sides and 196-318 µm on the unaffected sides (P = 0.400, Mann-Whitney U test).

**Table 1:** Demographic and clinical data M: male, F: female, L: left, R: right, TMH: tear meniscus height

	Case 1	Case 2	Case 3
Sex	M	F	M
Age (years)	66	82	53
Side	L	R	L
Type of anomaly	Dehiscence	Thinning	Thinning
TMH (µm)			
Affected side	616	464	209
Unaffected side	292	318	196

The dehiscent canaliculus was closed by sutures after making a raw surface and inserting a lacrimal tube. The thinned-out canalicular roof was covered using a conjunctival flap in the patient with punctal obstruction. There was no canalicular or nasolacrimal duct obstruction intraoperatively confirmed with probing in these two patients. However, recurrence was noted at two and five months after surgery. The other patient opted to not undergo any surgical intervention. Probing was not done in the non-surgical case due to possible rupture of the thin canalicular wall.

## Discussion

We report rare cases of canalicular wall anomaly affecting the lower canaliculus of three adult patients who presented with epiphora. The canalicular wall dehiscence and thinning presented physically resembled canalicular wall aplasia and hypoplasia described by Ali and Naik [2]. Based on their classification, one of our patients had focal single canalicular wall dehiscence, and the other two had focal single canalicular wall thinning [2].

Similar to the study of Ali and Naik [2], all our patients had epiphora as a presenting symptom, unilateral involvement of the lower canaliculus, and isolated single wall involvement, affecting only the roof. The consistent involvement of the lower canaliculus and its roof may be indicative of a specific vulnerability in this region. Our reports differ as our patients were older, had a later onset of symptoms, and had no systemic anomalies, and only one presented with concomitant punctal obstruction. The cases in the study of Ali and Naik [2] had an age range of 2-12 years, most noted epiphora since birth, two had systemic anomalies, and all had associated lacrimal disorders.

Epiphora may have become more evident with aging due to involutional changes such as caruncle and plica hypertrophy seen in one case [3,4]. Since other patent lacrimal structures provide alternative drainage, a single canalicular wall involvement may have caused no epiphora or some degree of epiphora that was not bothersome to our patients during their younger years. With this regard, late presentation of congenital canalicular wall dysgenesis was considered for our patients. However, since no previous ophthalmologic consultation was recorded, a congenital anomaly cannot be clearly established.

Other possible causes of acquired lacrimal obstruction were then explored, including infection, inflammation, neoplasm, mechanical obstruction, and trauma [5]. Chronic blepharitis, ocular cicatricial pemphigoid, Stevens-Johnsons syndrome, eyelid malposition, topical ophthalmic medications, and chemotherapy were reported to cause proximal lacrimal system obstruction [1,6,7]. Satchi and McNab [8] encountered cases of idiopathic acquired lacrimal canalicular obstruction, although none of their patients had canalicular wall anomalies. Our reported cases showed no predisposing factors that may cause the destruction of the canalicular wall. Canalicular fistulas and tears were also noted in post-trauma patients [9,10]. The dehiscent canaliculus reported had smooth borders with no post-traumatic signs [2]. Involutional changes of the canalicular wall due to the advanced age of our patients have been considered [11]. However, an involutional etiology would most likely present as a bilateral and symmetric disorder [5]. After a detailed history and thorough examination, no cause can be identified for all three cases.

The use of AS-OCT to measure TMH provided quantitative data for further understanding the functional implications of the canalicular wall anomaly [12]. Singh et al. [13] showed that TMH measured by OCT is statistically significant for a Munk severity grade of 2 and above. The lack of statistical significance between the affected and unaffected sides may be due to small statistical power from the small number of patients or suggests that our patients may have had relatively less severe epiphora. This may be attributed to monocanalicular involvement and lack of other lacrimal anomalies.

Management of canalicular wall anomaly has been focused on alleviating symptoms by primarily treating associated lacrimal disorders [2]. A case of multiple canalicular wall hypoplasia with incomplete punctal canalization that underwent membranotomy had good functional and anatomical outcomes after a mean follow-up of 28 months [14]. The long duration of symptoms in all our patients and the decision of one to forgo surgery raise uncertainties on the severity of epiphora and the benefits and risks of intervention. We then opted for a less invasive approach by simply suturing the dehiscent canaliculus and applying a conjunctival flap over the hypoplastic one. However, recurrences were noted at two and five months after surgery. A deeper muscular layer may have been involved and weakened the lacrimal pump mechanism. This was not adequately addressed by our choice of treatment.

## Conclusions

We report three cases of lacrimal canalicular wall dehiscence and thinning found in adults. Although the anomalies resembled canalicular wall aplasia and hypoplasia, a congenital etiology could not be clearly established due to the late presentation of symptom and lack of other congenital anomalies. Nonetheless, it suggests that lacrimal canalicular wall dehiscence and thinning may initially be asymptomatic and present with no associated lacrimal anomalies, leading to late diagnosis and intervention. Further investigations for underlying etiologies and anatomical variations are warranted to develop more effective diagnostic and treatment strategies for such disorders.
